# Detecting spatio-temporal hotspots of scarlet fever in Taiwan with spatio-temporal Gi* statistic

**DOI:** 10.1371/journal.pone.0215434

**Published:** 2019-04-16

**Authors:** Jia-Hong Tang, Tzu-Jung Tseng, Ta-Chien Chan

**Affiliations:** 1 Institute of Statistical Science, Academia Sinica, Taipei, Taiwan; 2 Research Center for Humanities and Social Sciences, Academia Sinica, Taipei, Taiwan; Kansas State University, UNITED STATES

## Abstract

A resurgence of scarlet fever has caused many pediatric infections in East Asia and the United Kingdom. Although scarlet fever in Taiwan has not been a notifiable infectious disease since 2007, the comprehensive national health insurance data can still track its trend. Here, we used data from the open data portal of the Taiwan Centers for Disease Control. The scarlet fever trend was measured by outpatient and hospitalization rates from 2009 to 2017. In order to elucidate the spatio-temporal hotspots, we developed a new method named the spatio-temporal Gi* statistic, and applied Joinpoint regression to compute the annual percentage change (APC). The overall APCs in outpatient and hospitalization were 15.1% (95% CI: 10.3%-20.2%) and 7.7% (95%CI: 4.5% -10.9%). The major two infected groups were children aged 5–9 (outpatient: 0.138 scarlet fever diagnoses per 1,000 visits; inpatient: 2.579 per 1,000 visits) and aged 3–4 (outpatient: 0.084 per 1,000 visits; inpatient: 1.469 per 1,000 visits). We found the counties in eastern Taiwan and offshore counties had the most hotspots in the outpatient setting. In terms of hospitalization, the hotspots mostly occurred in offshore counties close to China. With the help of the spatio-temporal statistic, health workers can set up enhanced laboratory surveillance in those hotspots.

## Introduction

Streptococcus pyogenes causes a variety of human diseases, including relatively mild skin infections as well as severe invasive diseases [1]. Among the diseases caused by this pathogen, scarlet fever, characterized by a sore throat, fever, headaches, swollen lymph nodes, and a characteristic rash, is predominantly an infectious disease of childhood, though it can also occur in older children and adults [2]. With improved nutrition and widespread use of antibiotics, scarlet fever is now a common, mild contagious disease. However, it is still a notifiable disease in many countries and regions. During the last decade, sporadic outbreaks and reemerging epidemics have been recorded worldwide, including in Vietnam [3], the Republic of Korea [4], China [5–7], Hong Kong [8, 9], Australia [10], Poland [11], and the United Kingdom [12, 13]. In Taiwan, scarlet fever was removed in 2007 from the list of notifiable diseases because of improved medical care capacities [14]. However, through using national health insurance data, the Taiwan Centers for Disease Control (Taiwan CDC) can still monitor the morbidity and hospitalization trends of scarlet fever.

Understanding the distribution of a disease in time and space is a foundation of public health. One of the most relevant analyses is related to analyzing the aggregation of disease cases in space, in time, or in both space and time [15–17]. In epidemiological studies, it is important to evaluate whether variation in the mortality and morbidity of a disease is randomly distributed or tends to occur as clusters over time and space in order to find the causative mechanism of the disease [18, 19]. Quantifying spatio-temporal patterns is important to identify the hotspots for public health intervention. Spatio-temporal statistics and tests are useful for adding precision to qualitative descriptions, facilitating the comparison of distributions, and drawing attention to some characteristics that might not be easily identified upon visual inspection.

The mapping of disease incidence and prevalence has long been a part of public health, epidemiology, and the study of disease in human populations [20]. Increasingly for epidemiological study, researchers have been looking for novel data visualization methods to aid in exploring spatial and temporal patterns. The ring map facilitates the visual assessment of multivariate spatial data by depicting individual datasets as separate rings of information surrounding a base map of a particular geographic region of interest [21, 22]. In this way, a ring map effectively summarizes multiple layers of data, presenting an array of regional attributes in a single spatially referenced graphic; ring maps have been used largely for representation of temporal data.

Local spatial statistics, such as local Moran’s I [23], the Getis-Ord *G*_i_* statistic [24, 25] and the score statistic [26], may assist with the identification of disease clusters. Local spatial statistics identify both those clusters with values higher in magnitude than are expected to be found by random chance, and the statistically significant patterns of high risk (hotspots) or low risk (cold spots) frequency locations. Such statistics require that a neighborhood can be defined around the location of interest. This is done by specifying weights for surrounding regions, and is tantamount to specification of the scale at which the local dependence or clustering is tested.

The Getis–Ord *G*_i_* hotspot cluster statistic is one of the popular approaches used for local spatial analysis. The *G*_i_* statistic measures the degree of spatial clustering of a local sample and how different it is from the expected value, which is the mean of the whole data set. Study of the annual expansion of disease clusters can use the annual maps of hotspots and follow their expansion. Yet, since the *G*_i_* statistic is a measure relative to the overall mean in a particular year, and since the mean varies annually, the discovery of temporally related hotspots is constrained.

This paper introduces a spatio-temporal *G*_i_* statistic whereby the spatial or temporal features that the user wants to incorporate in the formulation are directly accounted for in the generation of neutral models. The objective of this paper is to investigate the spatio-temporal patterns for scarlet fever in Taiwan and use the proposed spatio-temporal *G*_i_* statistic for the detection of local clusters and anomalies in outpatients and hospital admissions. A ring map using ArcPy and Python language to develop the ArcToolbox (http://www.esri.com/esri-news/arcuser/fall-2013/looking-at-temporal-changes) was created to visually explore the spatio-temporal distribution of scarlet fever using the proposed *G*_i_* Z-Scores on one map and also to identify the spatio-temporal hotspots [27].

## Materials and methods

### Ethics

The data we used in this study were all from the Taiwan CDC open data portal (https://data.cdc.gov.tw/en/). The values are all the aggregated counts of scarlet fever cases and total visits from outpatient and inpatient settings in each city or county. Consequently, approval from the institutional review board (IRB) was not required, and we also did not need to get consent from each patient.

### Data source

The Taiwan CDC open data portal, which can be accessed by the public, provides more than 250 datasets, including all notifiable disease cases and emergency department visits for selected syndromes, and information on quarantine practices, vaccines, nosocomial infections, and the like. Most of the information is updated automatically. More than 99% of the Taiwan population is covered by National Health Insurance (NHI). The Taiwan CDC receives aggregated numbers of outpatient, inpatient, and emergency room (ER) visits from NHI claims data via machine-to-machine interface, since 2008. In this study, yearly county-level data from scarlet fever cases in Taiwan were collected online during the period from 2009 to 2017, from Taiwan CDC’s open data portal. The scarlet fever dataset comprises numbers of inpatients and outpatients by gender and age group to provide an understanding of epidemic situations and disease trends in different cities and counties.

### Statistical methods

#### Temporal trend

We used Joinpoint Regression Software version 4.5.0.1, developed by the National Cancer Institute, to examine annual percentage changes (APC) in overall and age-specific outpatient and hospitalization rates from 2009 to 2017 [7]. A permutation test procedure in Joinpoint regression was used to assess whether an annual percentage change was significantly different from zero and the *p*-value for a two-sided test [28]. In describing trends, an increase or decrease is proved when the slope of APC is statistically significant (*p*<0.05). When the slope of annual percentage change is significant (p<0.05), it indicates an increasing or decreasing trend. A stable trend means a non-significant annual percentage change within the period (p≥0·05).

#### Spatio-temporal trend

In epidemiology, disease maps are often used to explore the hotspots of diseases [29, 30]. Disease incidence and prevalence contain both spatial and temporal attributes. One common approach to understanding spatial and temporal trends is to break the data up into a series of time snapshots. However, this usually leads to concerns of discontinuity.

The *G*_i_* statistic, introduced by Getis and Ord [24, 25], can be used as a measure of the degree of spatial clustering. Exploring the spatio-temporal pattern of the disease hotspots is limited with the *G*_i_* statistic since relative hotspots are accepted according to the distribution pattern of a specific period such as year, or month.

For the purpose of developing a modification of the *G*_i_* statistic which incorporates the spatio-temporal association and correlations, we consider both the spatial characteristic as well as the temporal characteristic in the formation of the spatio-temporal neighborhood. Common conceptualizations of spatial relationships include inverse distance, travel time, fixed distance, K nearest neighbors and contiguity. For the temporal characteristics, each observation at the current time and location is not only influenced by the previous time at the location, but also affected by the previous time of its spatial neighbors. A time lag is used to capture this feature. Then the spatial neighborhood and the time lag are combined to form the spatio-temporal neighborhood.

A modified *G*_i_* is developed in this study which identifies year-to-year hotspots which are relative to the past few years’ observations. The spatio-temporal *G*_i_* statistic is proposed as follows.

$$G_{i}^{*}(d,t)=\frac{\sum_{t=0}^{l}\sum_{j\in \partial i}w_{ij}(d,t)\cdot x_{jt}-\bar{x}\sum_{t=0}^{l}\sum_{j\in \partial i}w_{ij}(d,t)}{S\sqrt{\frac{n\sum_{t=0}^{l}\sum_{j\in \partial i}w_{ij}^{2}(d,t)-(\sum_{t=0}^{l}\sum_{j\in \partial i}w_{ij}(d,t){)}^{2}}{n-1}}}$$ (1)

where *x*_*j*_ is the specified attribute value of location *j*, ∂*i* denotes the set of spatial neighbors of location *i* (including location *i*), *l* is the designated time lag, *n* is the number of spatio-temporal neighbors of location *i* (including location *i*), *w*_*ij*_ (*d*, *t*) is the spatio-temporal weight for spatio-temporal neighbor *j* from location *i*, and $\bar{x}$ and *S* represents the mean and the standard deviation of the specified attribute of the set of spatio-temporal neighbors of location *i* (including location *i*) and where

$$S=\sqrt{\frac{\sum_{t=0}^{l}\sum_{j\in \partial i}x_{jt}^{2}}{n}-{\bar{x}}^{2}}$$ (2)

When *l* = 0, this modified *G*_i_* statistic is the same as the original form. Our notion of a spatio-temporal neighborhood is distinct from the traditional notions, since we consider both a spatial characterization as well as a temporal characterization of neighborhoods. To identify and describe spatio-temporal association between location *i* and its spatio-temporal neighbors, the weight function is defined as follows:

$$w_{ij}(d,t)=(1-\frac{d_{ij}}{\sum_{j\in \partial i}d_{ij}}{)}^{t+1},j\in \partial i,$$ (3)

where *d*_*ij*_ is the distance between locations *i* and *j*, and *t* represents time lags.

The proposed weight function is a kind of distance declining effect that decreases exponentially with time lags. The distance declining effect means that as distance increases from a particular location, the weight between spatio-temporal neighbors lessens. We use Z scores to measure the standard deviation; under the normal distribution, scores within one standard deviation from the average value will account for 68.27% of the data, those within two standard deviations will reach 95.45%, and those within three standard deviations will reach 99.73%.

Due to the spatio-temporal structure of the data, neighboring locations exist both in time and in space. In this study, the first-order Queens Case Contiguity method [31] was used to define neighborhood size in space. To define temporal neighbors, time-lagged correlations, which describe how similar the time series is with itself, were calculated and tested until the first statistically insignificant result appeared. The results of time lag selection in this study are shown inS1 and S2 Tables.

The R source code and the data we used are provided at the open repository, figshare.com (https://figshare.com/s/cf8762f07e48ec79a4df). The ring map and associated Taiwan base map created in this study are also at figshare.com (https://figshare.com/s/8d2a3887482079d3e2bd).

## Results

### Epidemiological trend of scarlet fever

In view of the Joinpoint regression results for the outpatient rate in Table 1, there was a significant upward trend of 15.1% (95% CI: 10.3%-20.2%) per year for the overall scarlet fever outpatient rate between 2009 and 2017. From the point of view of age-specification, APCs of the scarlet fever outpatient rate increased 12.4% (95% CI: 8.8%-16.2%), 17.9% (95% CI: 11.7%-24.3%), 21.5% (95% CI: 15.2%-28.1%), and 19.8% (95% CI: 13.3%-26.6%) per year from 2009 to 2017 for for the age groups 0–2, 3–4, 5–9 and 10–14 years, respectively. For the age group 15+, APC of the scarlet fever outpatient rate was stable between 2009 and 2017. For the outpatient rates of the age group 5–9 years, there was higher deviation than for the other age groups.

**Table 1:** Descriptive statistics on annual rate of scarlet fever in outpatient setting from 2009 to 2017 and annual percentage change (APC).

Descriptive Statistics						
OPD Age group	N1	Rate Range	Mean (per 1,000)	Std. Deviation	N2	APC % (95%CI)
0–2	198	(0, 0.4)	0.0315	0.0446	9	12.4 (8.8, 16.2)
3–4	198	(0, 2.02)	0.0836	0.17327	9	17.9 (11.7, 24.3)
5–9	198	(0, 4.02)	0.138	0.30878	9	21.5 (15.2, 28.1)
10–14	198	(0, 0.23)	0.0262	0.03283	9	19.8 (13.3, 26.6)
15+	198	(0, 0.03)	0.0005	0.00237	9	3.1 (-1.5, 7.9)
Overall	198	(0, 0.18)	0.0120	0.01593	9	15.1 (10.3, 20.2)

Abbreviations: CI, confidence interval. Age is in years. N1 represents annual rate in 22 cities or counties for 9 years. N2 represents annual rate in the whole of Taiwan for 9 years.

In the light of the Joinpoint regression results for the inpatient rate in Table 2, there was a significant upward trend of 7.7% (95% CI: 4.5%-10.9%) per year for the overall scarlet fever inpatient rate between 2009 and 2017. For the age-specific perspective, APCs of the scarlet fever inpatient rate increased 12.8% (95% CI: 7.2%-18.6%), 16.0% (95% CI: 9.5%-22.9%) and 16.3% (95% CI: 9.4%-23.4%) per year from 2009 to 2017 for the age groups 3–4, 5–9 and 10–14, respectively. For the age groups 0–2 and 15+, APCs of the scarlet fever inpatient rate were stable between 2009 and 2017. For inpatient rates of the age groups 3–4 and 5–9, there were higher deviations than for the other groups. This shows that inpatient rates between 2009 and 2017 of the age groups 3–4 and 5–9 were more spread out and had larger ranges.

**Table 2:** Descriptive statistics on annual rate of scarlet fever in inpatient setting from 2009 to 2017 and annual percentage change (APC).

Descriptive Statistics						
Hospitalization Age group	N1	Rate Range	Mean (per 1,000)	Std. Deviation	N2	APC% (95%CI)
0–2	198	(0, 3.24)	0.5097	0.66963	9	3 (-2.7, 9.1)
3–4	198	(0, 55.56)	1.4694	4.23467	9	12.8 (7.2, 18.6)
5–9	198	(0, 83.33)	2.5785	6.18071	9	16.0 (9.5, 22.9)
10–14	198	(0, 7.84)	0.6785	1.21436	9	16.3 (9.4, 23.4)
15+	198	(0, 0.05)	0.002	0.00695	9	8 (-7.5, 26.2)
Overall	198	(0, 4.02)	0.1265	0.32537	9	7.7 (4.5, 10.9)

Abbreviations: CI, confidence interval. Age is in years. N1 represents annual rate in 22 cities or counties for 9 years. N2 represents annual rate in the whole of Taiwan for 9 years.

**Fig 1:**
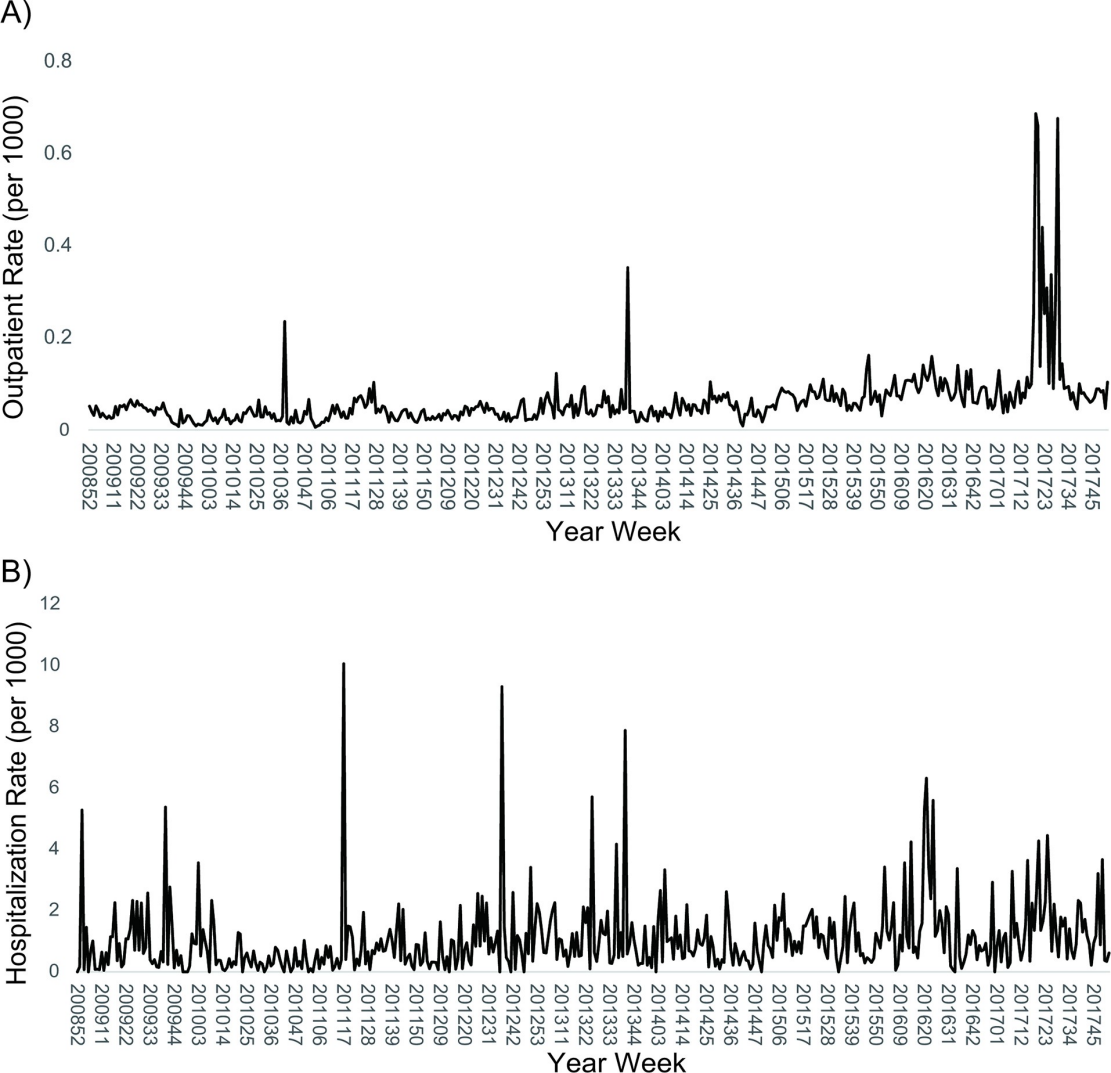
Temporal trend of scarlet fever, 2009 to 2017. (A) Outpatient visit rate. (B) Hospitalization rate.

The temporal analysis showed that there were three peaks of scarlet fever outpatient cases during the study period. The first small peak occurred in October 2010, the second peak (2013) also began in October, and the highest level was from May to July 2017 (Fig 1A). The average hospitalization rate usually peaked in April, June and September (Fig 1B). The highest three hospitalization rates occurred in April 2011, September 2012 and September 2013.

### Spatio-temporal analysis

**Fig 2:**
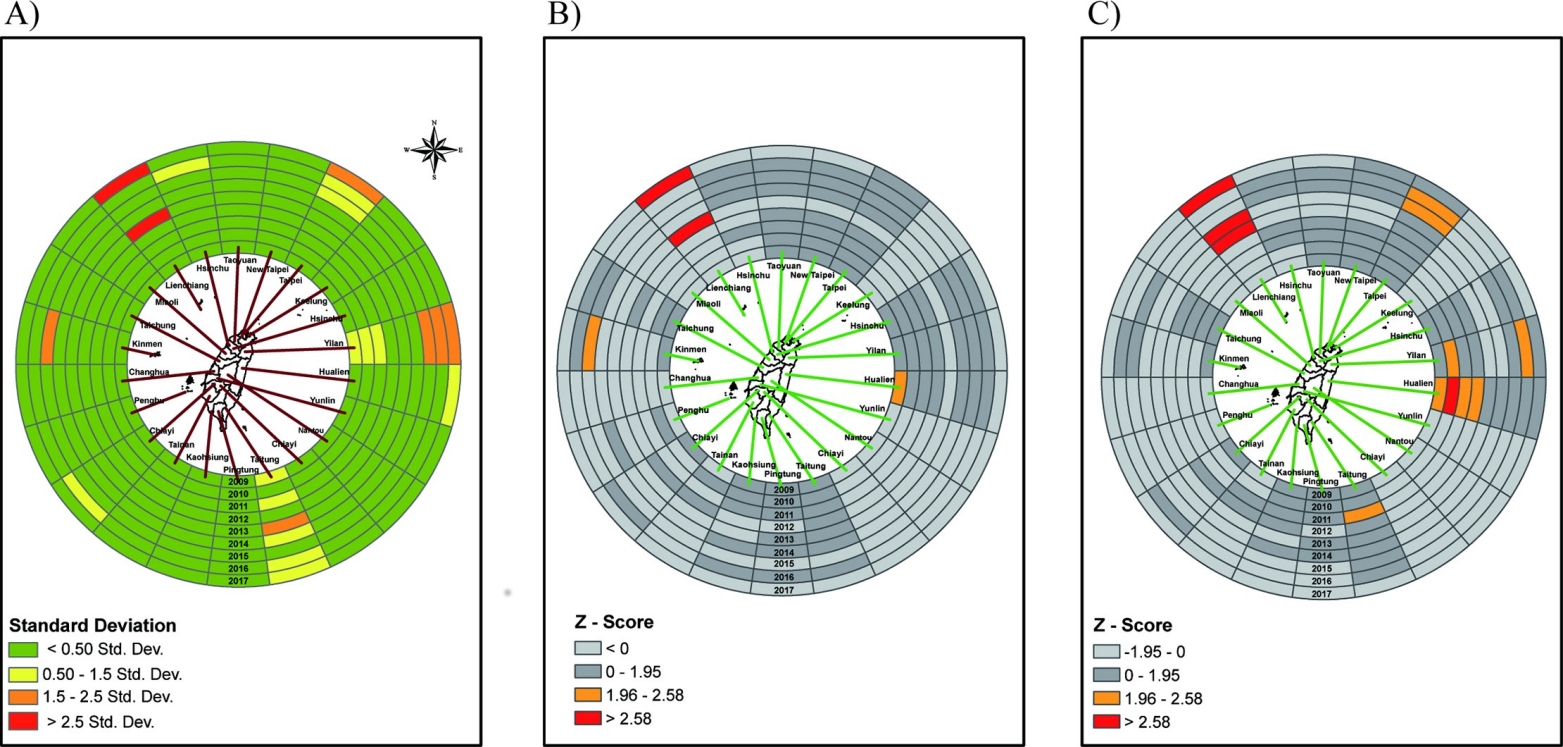
Ring map of outpatient visit rate of scarlet fever in age group 3–4 years. (A) A Z score ring map from innermost in 2009 to outermost in 2017. (B) A ring map of Z-scores by applying spatio-temporal *G*_i_* without time lag. (C) A ring map of Z-scores by applying spatial-temporal *G*_i_* with 1 year lag.

**Fig 3:**
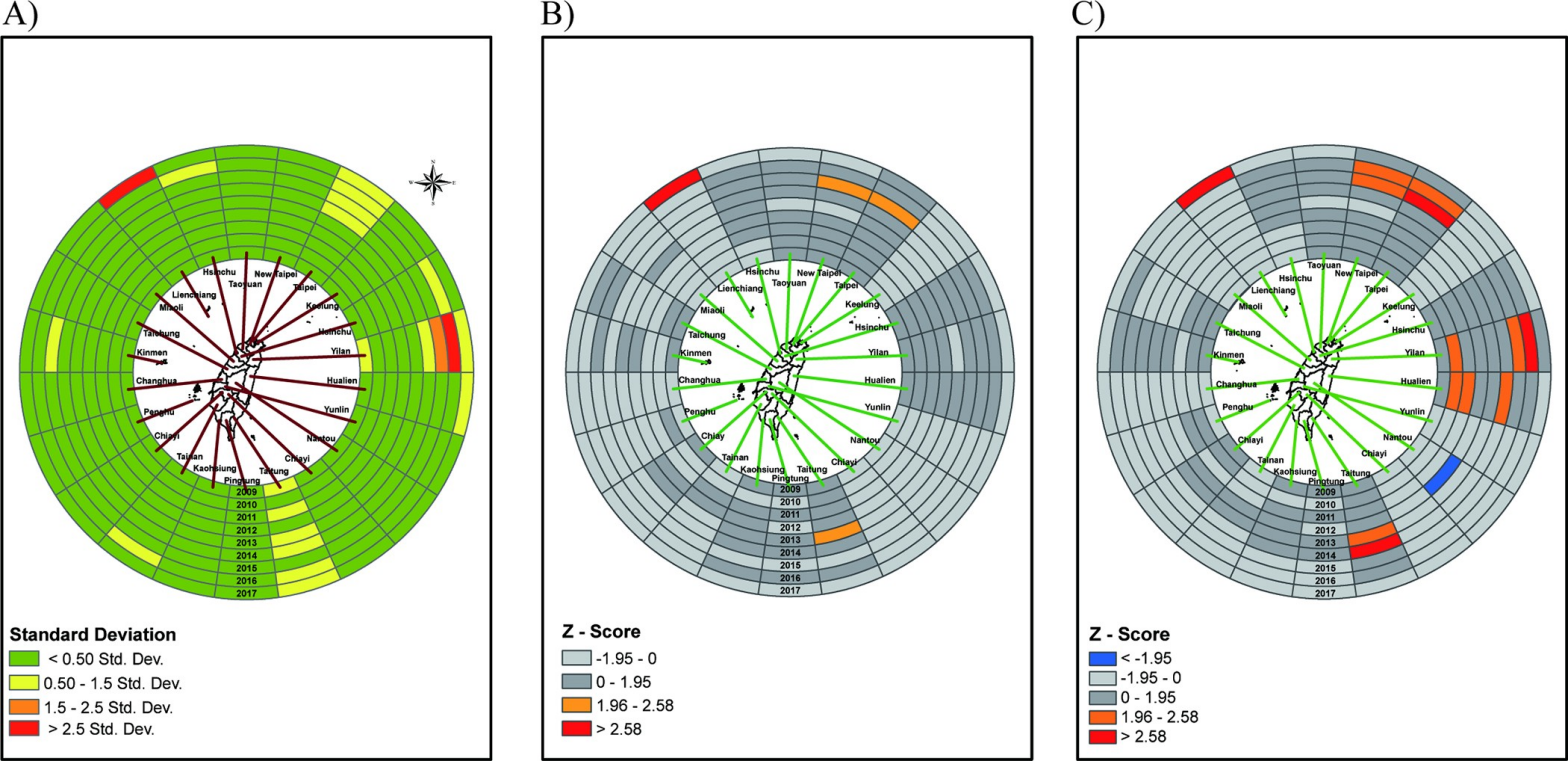
Ring map of outpatient visit rate of scarlet fever in age group 5–9 years. (A) A Z score ring map from innermost in 2009 to outermost in 2017. (B) A ring map of Z-scores by applying spatio-temporal *G*_i_* without time lag. (C) A ring map of Z-scores by applying spatial-temporal *G*_i_* with 1 year lag.

The ring map of the outpatient visit rate is presented as a Gi* result for patients 3 to 4 years of age. Fig 2A shows Z scores of standard deviation in outpatient visits by four age groups. We use Z scores to measure the distance from the mean in standard deviations; under the normal distribution, scores within one standard deviation from the average value account for 68.27% of the data, those within two standard deviations reach 95.45%, and those within three standard deviations reach 99.73%. The red color in the map means a Z score higher than 2.5 standard deviations away from the mean. Lienchiang County (upper left corner) is the primary hot spot, with a Z score greater than 2.5 standard deviations. Yilan County, Kinmen County and Taipei City are the secondary hotspots, with Z scores between 1.5 to 2.5 standard deviations. In contrast, the crude rate of this age group showed several hotspots before standardization (S1 Fig). In addition, in terms of spatio-temporal Gi* without time lag, only Lienchiang County was a hotspot (Fig 2B). Regarding spatio-temporal Gi* with a 1-year lag, it was discovered that there were significant hotspots in Lienchiang County and Hualien County (Fig 2C). This means that Lienchiang has a significant, high outpatient visit rate in the age 3 to 4 years old group. Among outpatients aged 5 to 9 years old, there are two hotspots identified with Z scores higher than 2.5 standard deviations, located in Lienchiang County and Yilan County (Fig 3A). In contrast, for the crude rate of this age group, there were higher outpatient rates in northern and eastern Taiwan (S2 Fig).

Next we found a hotspot in Lienchiang County by spatio-temporal Gi* without lag year analysis (Fig 3B). After using spatio-temporal Gi* with a 1-year lag, Lienchiang County, Yilan County and Taitung County are revealed to be significant hotspots (Fig 3C).

**Fig 4:**
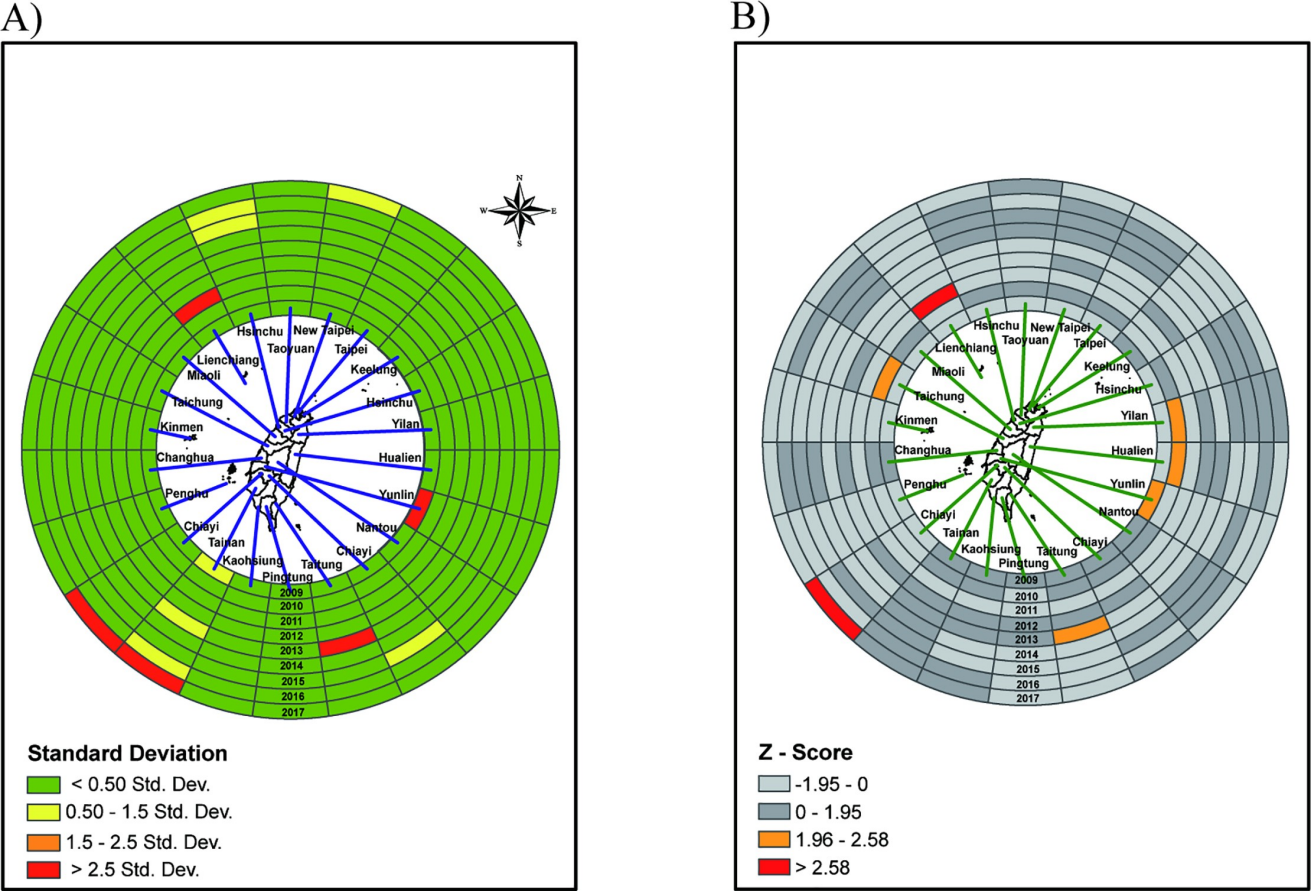
Ring map of hospitalization rate of scarlet fever in age group 3–4 years. (A) A Z score ring map from innermost in 2009 to outermost in 2017. (B) A ring map of Z-scores by applying spatio-temporal *G*_i_* without time lag.

**Fig 5:**
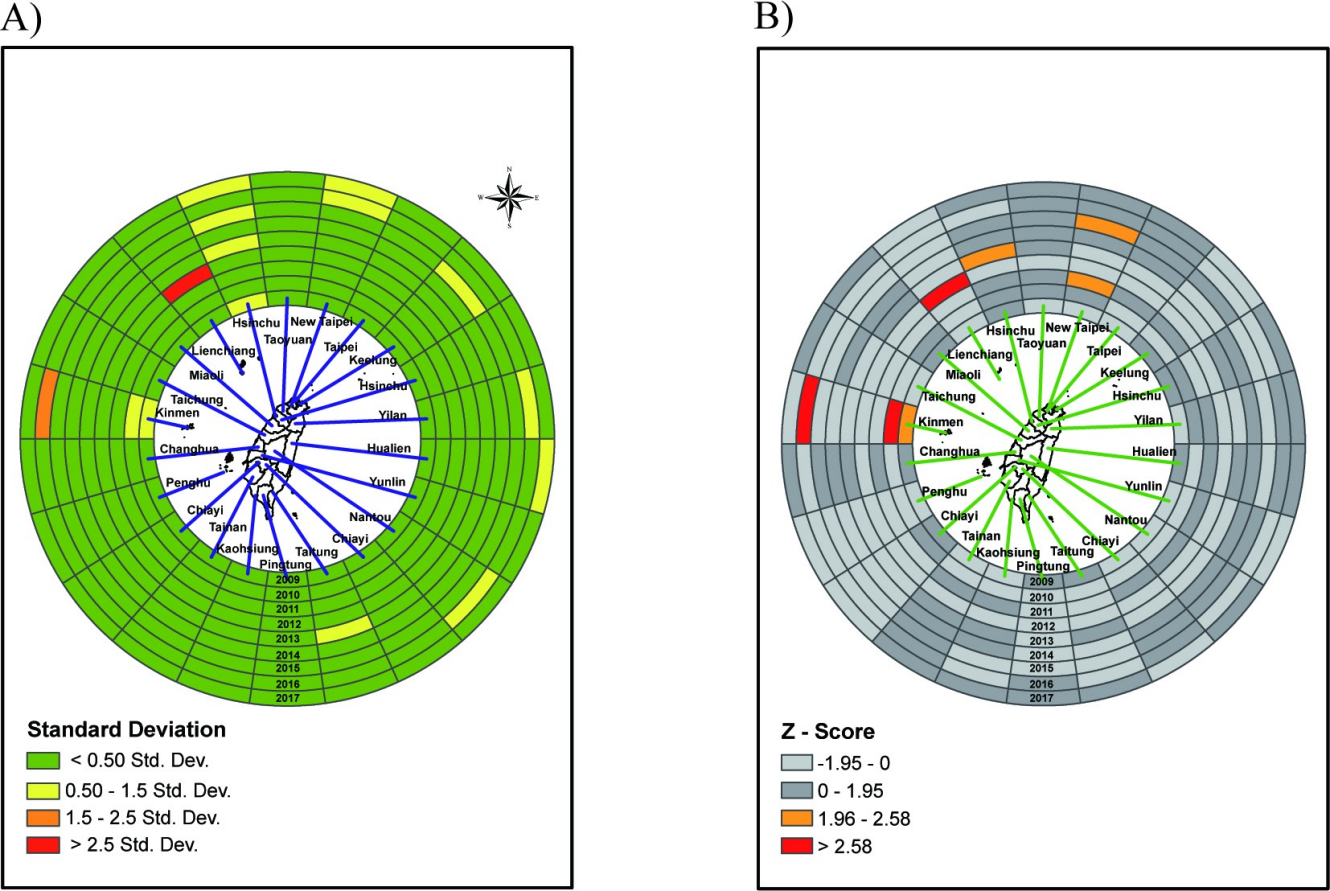
Ring map of hospitalization rate of scarlet fever in age group 5–9 years. (A) A Z score ring map from innermost in 2009 to outermost in 2017. (B) A ring map of Z-scores by applying spatio-temporal *G*_i_* without time lag.

Regarding hospitalization for scarlet fever from 3 to 4 years of age, Yilan County in 2009, Lienchiang County in 2011, Taitung County in 2013, and Tainan and Chiayi City in 2017 are hotspots with Z scores higher than 2.5 standard deviations (Fig 4A). However, compared to the standardized rates, the crude rates were high in Lienchiang County, New Taipei City, Nantou County and Tainan City (S3 Fig). To consider the spatial neighbor relationship, spatio-temporal Gi* was used to find hotspots. We found that hotspots among those aged 3 to 4 were Chiayi City and Lienchiang County (Fig 4B). After the analysis of spatio-temporal Gi* with time lag, there was no significant difference between the spatial and spatio-temporal Gi* in the ring map. For the 5- to 9-year-old age group, Z scores in Lienchiang County, higher than 2.5 standard deviations, and Kinmen, with 1.5 to 2.5 standard deviations, represent primary and secondary hotspots, respectively (Fig 5A). In contrast, there were higher crude rates located in northern Taiwan (S4 Fig). After applying spatio-temporal *G*_i_* to find hotspots, we found that hotspots were Kinmen County and Lienchiang County on offshore islands, and there was no significant difference between the spatio-temporal Gi* with or without time lag in the ring map (Fig 5B).

## Discussion

In this article, we have proposed a new spatio-temporal Gi* statistic to cope with the question associated with the Gettis-Ord Gi* statistic where the time-to-time autocorrelation of spatio-temporal data could not be taken into account in hotspot detection. Many hotspot detection approaches first perform a spatial characterization of the data then find the temporal pattern. The work presented in this paper sets itself apart from other studies by finding temporal intervals in the dataset. The temporal neighborhood was defined as a certain time window which is determined by the number of consecutive time-lagged significant correlation coefficients. Then a modified weighted function in the proposed spatio-temporal Gi* statistic was used to precisely reflect the correlation between space and time in the data.

Mapping the distribution of diseases is a common exercise, and time and space are important factors which affect the spread of infectious diseases. Therefore, in addition to visualizing the outpatient visit rates and hospitalization rates for scarlet fever with a ring map GIS package, this study uses spatio-temporal *G*_i_* to present spatial and temporal scarlet fever hotspots. The information helps us understand where the hotspots are, information which needs to be taken into account by both scholars and policy makers.

We have provided a new approach to show scarlet fever hotspots in Taiwan by using ring maps and a novel spatio-temporal *G*_i_* statistic. The annual percentage changes in outpatient rate and hospitalization rate were 15.1% and 7.7% during 2009 to 2014. The highest two infected groups were children aged 3–5 and 5–9 years in our research. The main epidemic peaks were from March to May, and the high-risk population was 5–6 years old [32]. Likewise, a previous study by Mahara, et al. [5] reported that the average incidence (83.8%) was among children 3–8 years old. Additionally, several studies in China (including Hong Kong) and South Korea found a higher incidence among children 4–7 years of age. Another study in the United Kingdom reported that 87% of cases were in children under 10 years of age, while the Department of Health, UK found that scarlet fever was most common between the ages of 2 and 8 years [12, 33].

The temporal analysis in our study found that outpatient visits peaked in September and May, while the peak of hospitalization occurred in April and October. Similar findings were reported by Wu et al. and Liang et al. in Taiwan, who showed that the peaks occurred in March to July [14, 32]. Incidence has also been reported to peak from March to June with a small second peak from November to January in China [34], from March to May in the UK [35], and during winter months in Korea [36].

The seasonal peak has varied in different regions, which might be attributable to climate, population density, geographic distribution and other public health factors [37]. As shown above, transmission of scarlet fever tends to occur more in spring and winter compared to other seasons.

A previous study in Taiwan had similar results from 2000 to 2006, and also reported that the cases of scarlet fever in Hualien and Taitung Counties in eastern Taiwan increased annually during that period [32]. In our study, the main epidemic peak was from March to May. Also, based on the percentage of outpatient visits in our study, cases in Yilan, Hualien and Taitung Counties are still increasing yearly from 2009 to 2017. Based on the original hospitalization rates, cases in Yilan and Hualien Counties (eastern Taiwan) were high, and the epidemic in Tainan City (western Taiwan) was even more pronounced. Scarlet fever hotspots occurred not only in eastern but also in western Taiwan and surrounding islands. Convenient transportation obviously lowers the geographical barriers to the spread of this infectious disease.

During our study period, scarlet fever, measured both in outpatient visits and the hospitalization rate, increased year by year. Although scarlet fever can be treated with drugs, serious complications such as toxic shock syndrome may occur if drugs are not used in time. In addition, sepsis and death are serious outcomes which bring great disease burden [38]. Nowadays, scarlet fever has been removed from Taiwan’s notifiable infectious diseases list. However, scarlet fever cases have resurged in the UK and China (including Hong Kong) in recent years, without any real cause being found [39]. By monitoring the outpatient and hospitalization rates of scarlet fever in Taiwan, we also found a significant increasing trend in recent years. It is recommended that the genetic evolution of group A Streptococcus (GAS) and multi-drug resistance be closely monitored [3].

The proposed spatio-temporal Gi* statistic was illustrated with yearly county-level data from scarlet fever cases in Taiwan during 2009 to 2017. The retrospective space-time scan statistic by SaTScan (https://www.satscan.org/) was applied for age groups 3–4 and 5–9 years, and results are shown in the Supplement (S5–S8 Figs). Compared with the proposed spatio-temporal Gi* statistic, the retrospective space-time scan statistic in the age group 3–4 and 5–9 years has fewer noticeable hotspots and clusters.

In addition, computing time depends highly on the size of the dataset. With our data, the calculation procedure takes 20 seconds to run on an Intel Core i5-8400 (2.8 GHz) computer. The data used in this study have been aggregated from the record level to county level, which ensures the identities of individuals cannot be determined by a reasonably foreseeable method. For record-level data or a real-time disease surveillance system, a space-time domain decomposition approach for parallel computation can reduce the effort required to identify hotspot patterns [40].

All spatial clustering approaches, regardless of their theoretical underpinning, statistical foundation, or mathematical specification, have limitations in accuracy, sensitivity, and the computational effort required for identifying clusters. As a result, a major challenge in practice is determining which technique will provide the most meaningful insights for a particular issue or dataset. The analytical results in this study have two limitations. First, our results are sensitive to spatio-temporal neighborhood size. As neighborhood size increases, hotspots will become larger and fewer; smaller neighborhood sizes capture more localized trends. There is inevitably an element of subjectivity in choosing an appropriate size for both spatial and temporal neighborhoods. To determine the appropriate size for both spatial and temporal neighborhoods, systematic data quality checks and analytic adjustments are needed. The choice of spatial neighborhood should reflect inherent relationships or characteristics of data. Once the spatial neighborhood is designated, the temporal neighborhood is constructed by discovering the similarity of autocorrelation. Second, scarlet fever has not been listed as a notifiable infectious disease in Taiwan since 2007, so some mild cases may have gone unreported.

## Conclusion

A spatial-temporal Gi* statistic was proposed in this paper to detect hotspots in the space-time domain. A ring map was used to summarize the Z scores calculated by the spatial-temporal Gi* statistic and to present an array of regional attributes in a single spatio-temporal reference graphic.

In conclusion, our study provides a new approach for better understanding of the spatio-temporal patterns of scarlet fever in Taiwan from 2009 to 2017. First, the increases in hotspots were mostly distributed in rural and offshore islands, especially in Hualien, Lienchiang and Jinmen Counties. For offshore islands, scarlet fever may be imported from China because of frequent contact and geographic proximity. Second, children under 9 years of age were more sensitive to scarlet fever infection, especially those aged 5–9. With the help of the spatio-temporal Gi* statistic, public health officials can identify the high risk areas, which will help them monitor scarlet fever epidemics more effectively and reduce the disease burden among children.

## Supporting information

S1 Fig Annual outpatient visit rate from innermost ring in 2009 to outermost ring in 2017 for age 3–4. (DOCX)

S2 Fig Annual outpatient visit rate from innermost ring in 2009 to outermost ring in 2017 for age 5–9. (DOCX)

S3 Fig Annual hospitalization rate from innermost ring in 2009 to outermost ring in 2017 for age 3–4. (DOCX)

S4 Fig Annual hospitalization rate from innermost ring in 2009 to outermost ring in 2017 for age 5–9. (DOCX)

S5 Fig Annual outpatient visit rate from innermost ring in 2009 to outermost ring in 2017 by SaTScan for age 3–4. (DOCX)

S6 Fig Annual outpatient visit rate from innermost ring in 2009 to outermost ring in 2017 by SaTScan for age 5–9. (DOCX)

S7 Fig Annual hospitalization rate from innermost ring in 2009 to outermost ring in 2017 by SaTScan for age 3–4. (DOCX)

S8 Fig Annual hospitalization rate from innermost ring in 2009 to outermost ring in 2017 by SaTScan for age 5–9. (DOCX)

S1 Table The lag selection of outpatient visits. (DOCX)

S2 Table The lag selection of hospitalizations. (DOCX)
