# Supplementary figures and images for: Detecting spatio-temporal hotspots of scarlet fever in Taiwan with spatio-temporal Gi* statistic
Source: PLoS One. 2019 Apr 16;14(4):e0215434. doi: 10.1371/journal.pone.0215434 (PMC6467404; doi:10.1371/journal.pone.0215434)

**S1 Fig.** Annual outpatient visit rate from innermost ring in 2009 to outermost ring in 2017 for age 3-4.


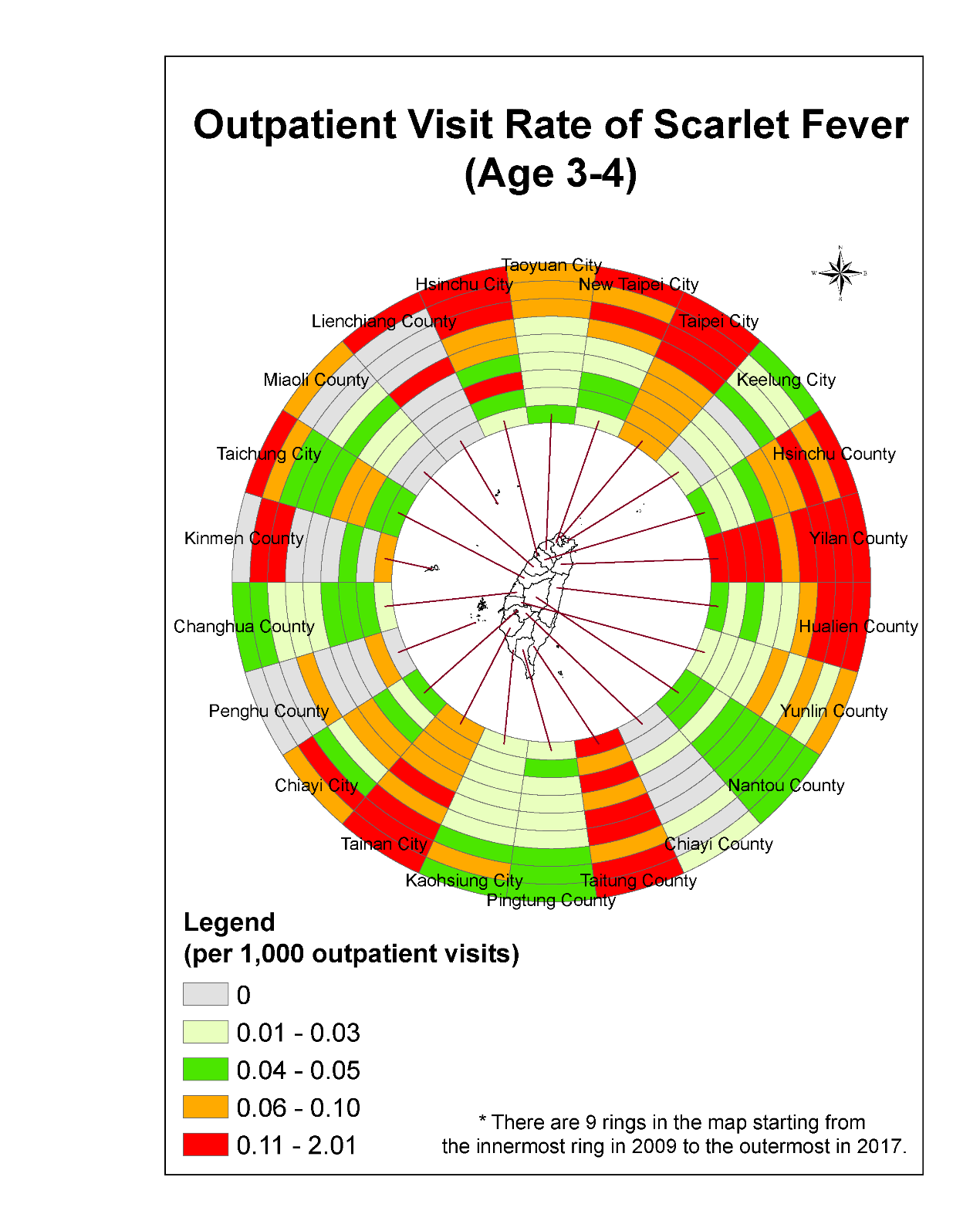

Supplement: S1 Fig — (DOCX) [file pone.0215434.s001.docx]

**S2 Fig.** Annual outpatient visit rate from innermost ring in 2009 to outermost ring in 2017 for age 5-9.


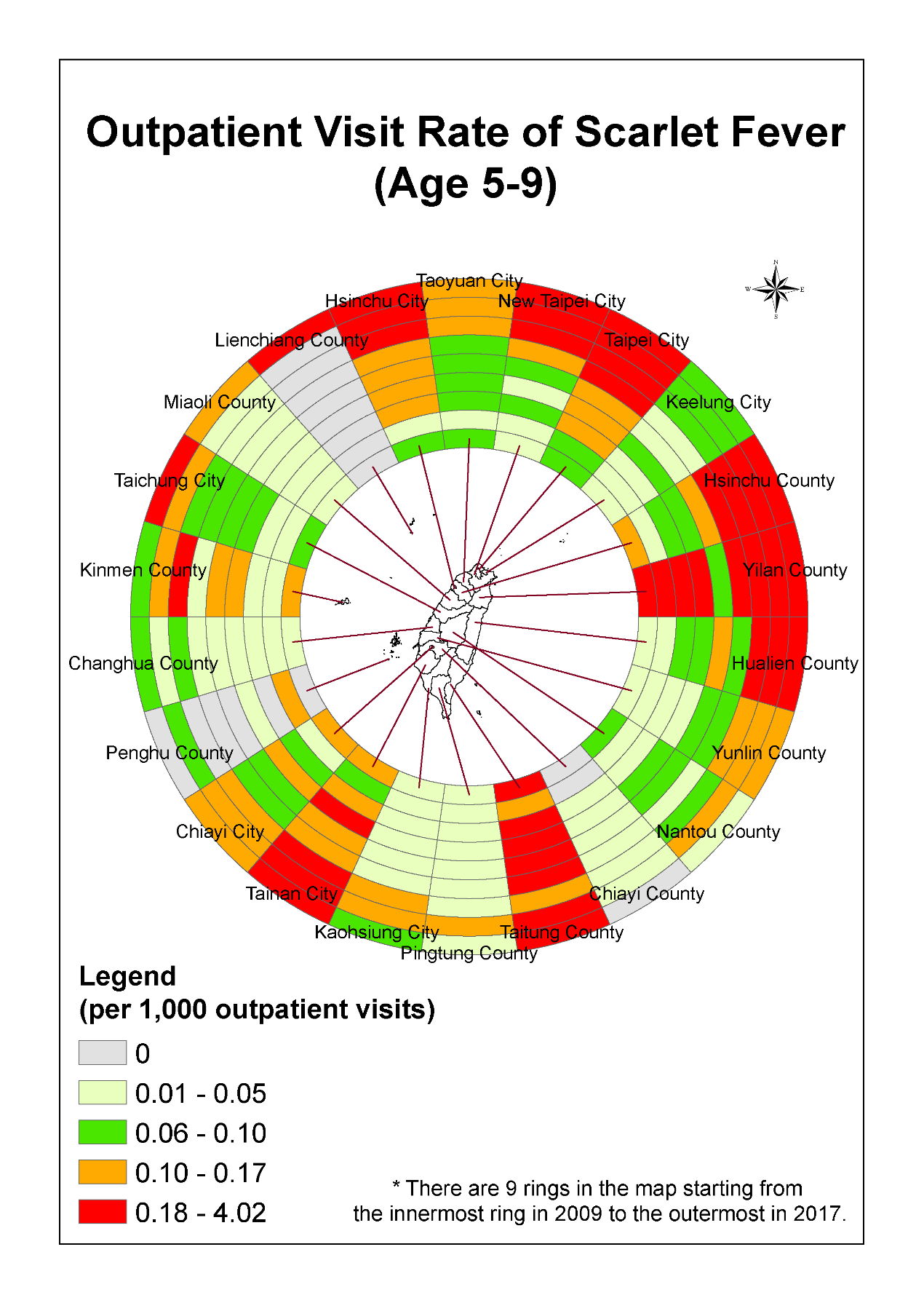

Supplement: S2 Fig — (DOCX) [file pone.0215434.s002.docx]

**S3 Fig.** Annual hospitalization rate from innermost ring in 2009 to outermost ring in 2017 for age 3-4.


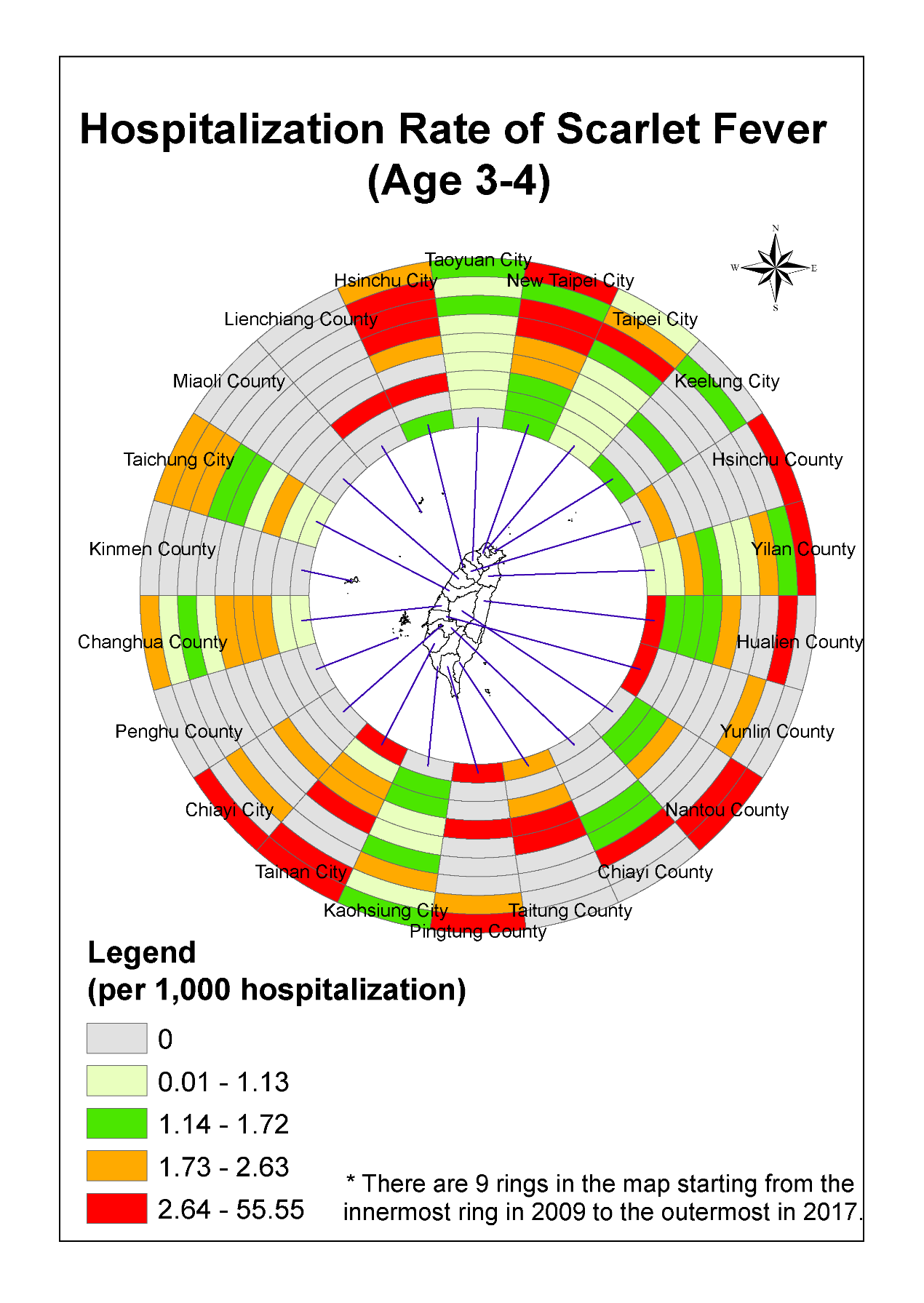

Supplement: S3 Fig — (DOCX) [file pone.0215434.s003.docx]

**S4 Fig.** Annual hospitalization rate from innermost ring in 2009 to outermost ring in 2017 for age 5-9.


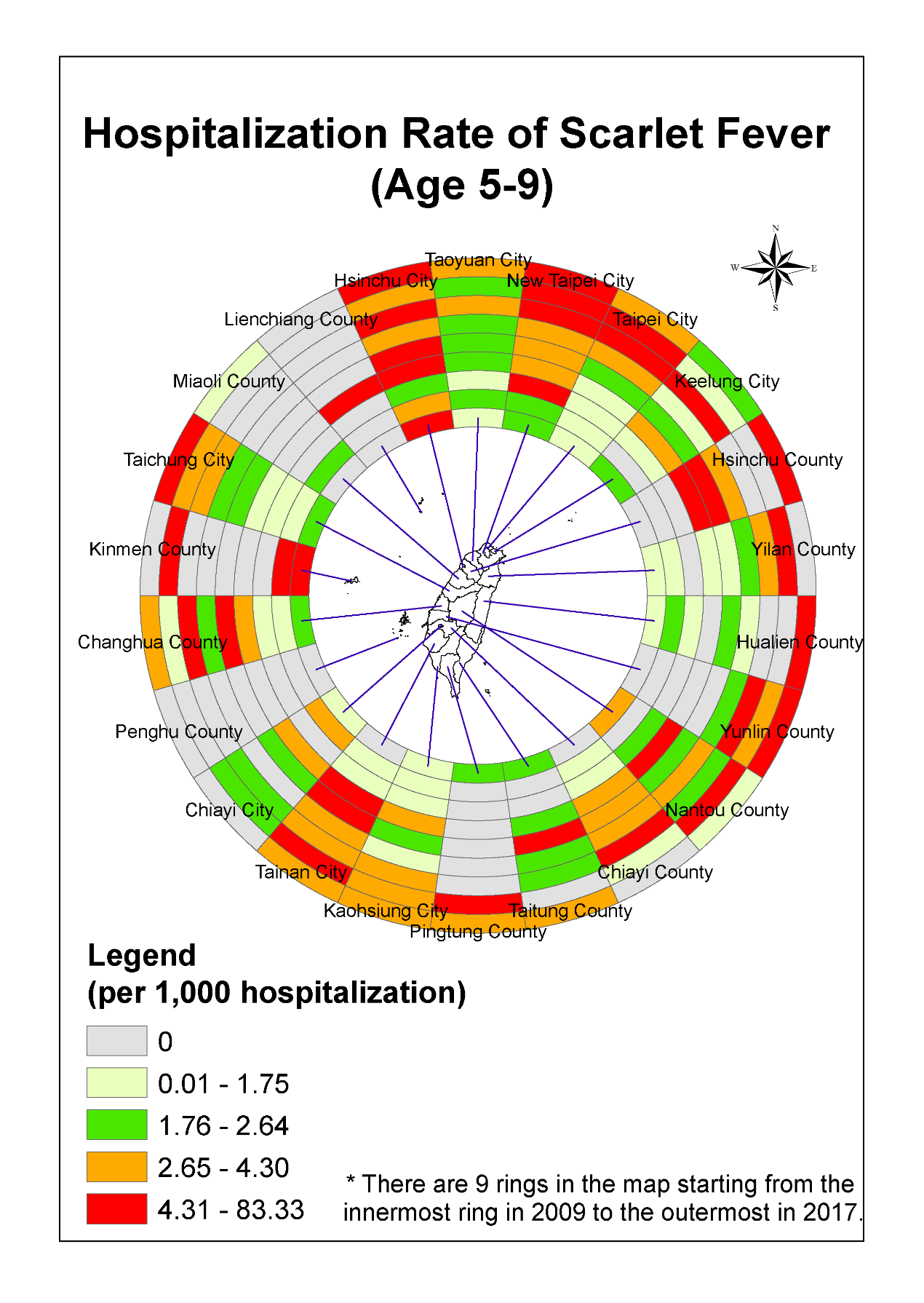

Supplement: S4 Fig — (DOCX) [file pone.0215434.s004.docx]
