# Supplementary material for: Detecting spatio-temporal hotspots of scarlet fever in Taiwan with spatio-temporal Gi* statistic
Source: PLoS One. 2019 Apr 16;14(4):e0215434. doi: 10.1371/journal.pone.0215434 (PMC6467404; doi:10.1371/journal.pone.0215434)

**S5 Fig.** Annual outpatient visit rate from innermost ring in 2009 to outermost ring in 2017 by SaTScan for age 3-4.


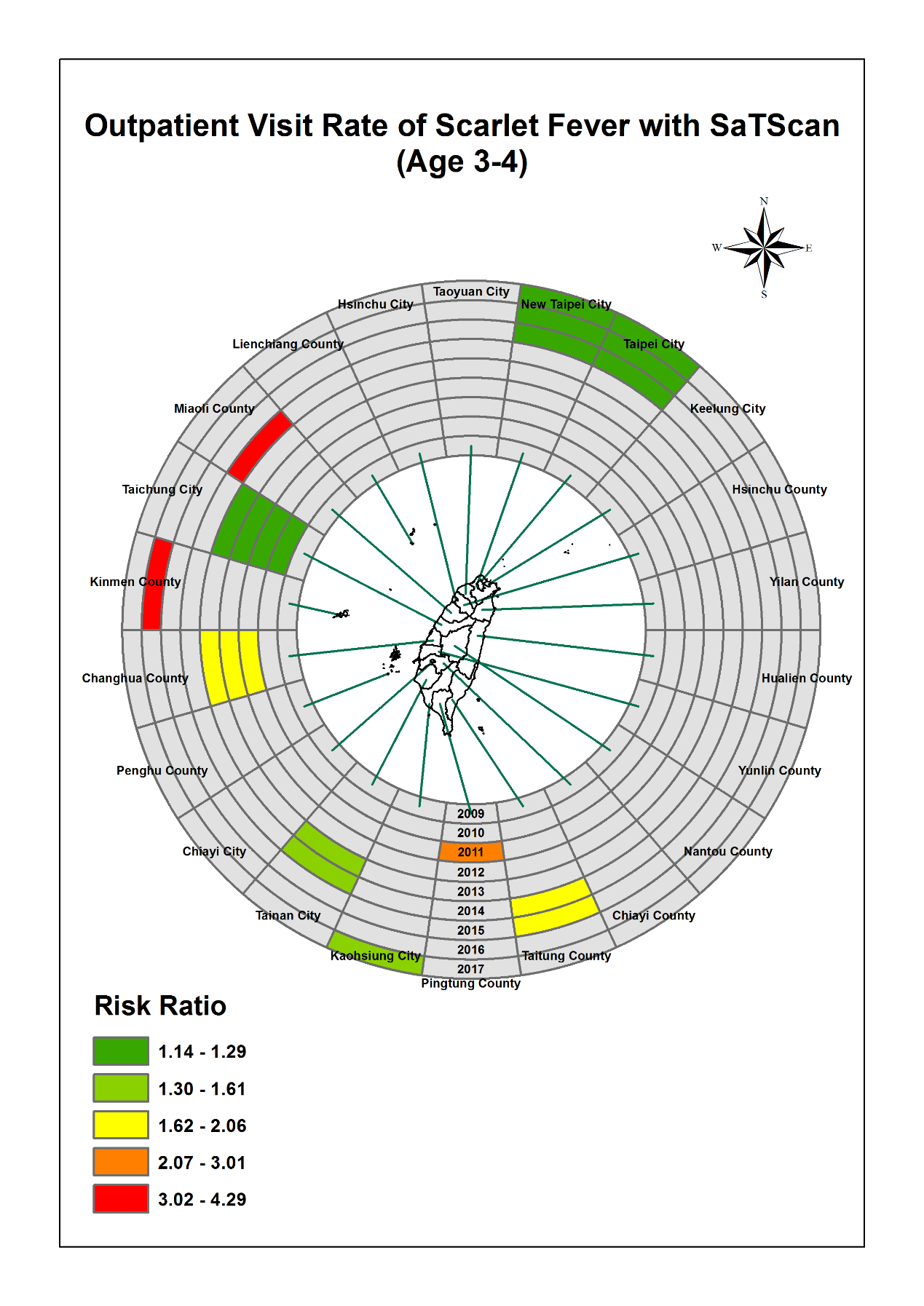

Supplement: S5 Fig — (DOCX) [file pone.0215434.s005.docx]
