# Supplementary material for: Detecting spatio-temporal hotspots of scarlet fever in Taiwan with spatio-temporal Gi* statistic
Source: PLoS One. 2019 Apr 16;14(4):e0215434. doi: 10.1371/journal.pone.0215434 (PMC6467404; doi:10.1371/journal.pone.0215434)
Supplement: S1 Table — (DOCX) [file pone.0215434.s009.docx]

**S1 Table.** The lag selection of outpatient visits

| Age group | 0-2 | | 3-4 | | 5-9 | | 10-14 | | 15+ | |
| --- | --- | --- | --- | --- | --- | --- | --- | --- | --- | --- |
| Time lag | Cor. Value | P-value | Cor. value | P-value | Cor. value | P-value | Cor. value | P-value | Cor. value | P-value |
| 0 | 1.000 |  | 1.000 |  | 1.000 |  | 1.000 |  | 1.000 |  |
| 1 | 0.236 | 0.002 | 0.164 | 0.030 | 0.261 | 0.015 | 0.219 | 0.035 | -0.011 | 0.890 |
| 2 | 0.169 | 0.037 | 0.107 | 0.185 | 0.123 | 0.075 | 0.140 | 0.182 | 0.041 | 0.611 |
| 3 | 0.311 | 0.000 | 0.064 | 0.463 | 0.181 | 0.098 | 0.125 | 0.347 | 0.000 | 1.000 |
| 4 | 0.107 | 0.330 | 0.781 | 0.000 | 0.143 | 0.136 | 0.133 | 0.326 | 0.010 | 0.918 |
| 5 | 0.111 | 0.304 | 0.212 | 0.047 | 0.173 | 0.107 | 0.060 | 0.538 | -0.037 | 0.732 |
| 6 | 0.097 | 0.439 | 0.179 | 0.151 | 0.131 | 0.295 | 0.046 | 0.612 | -0.053 | 0.675 |
| 7 | -0.060 | 0.700 | 0.143 | 0.356 | 0.102 | 0.512 | 0.055 | 0.681 | -0.080 | 0.605 |
| 8 | -0.039 | 0.864 | -0.043 | 0.850 | -0.083 | 0.712 | 0.089 | 0.706 | 0.273 | 0.219 |
| Selected lag length | 3 | | 1 | | 1 | | 1 | | 0 | |

All tests were significant at the 0.05 level.
