# Supplementary material for: Detecting spatio-temporal hotspots of scarlet fever in Taiwan with spatio-temporal Gi* statistic
Source: PLoS One. 2019 Apr 16;14(4):e0215434. doi: 10.1371/journal.pone.0215434 (PMC6467404; doi:10.1371/journal.pone.0215434)
Supplement: S2 Table — (DOCX) [file pone.0215434.s010.docx]

**S2 Table.** The lag selection of hospitalizations

| Age group | 0-2 | | 3-4 | | 5-9 | | 10-14 | | 15+ | |
| --- | --- | --- | --- | --- | --- | --- | --- | --- | --- | --- |
| Time lag | Cor. value | P-value | Cor. value | P-value | Cor. value | P-value | Cor. value | P-value | Cor. value | P-value |
| 0 | 1.000 |  | 1.000 |  | 1.000 |  | 1.000 |  | 1.000 |  |
| 1 | 0.310 | 0.000 | -0.013 | 0.859 | -0.017 | 0.821 | 0.171 | 0.023 | -0.011 | 0.890 |
| 2 | 0.185 | 0.022 | -0.029 | 0.725 | -0.032 | 0.698 | 0.015 | 0.853 | 0.041 | 0.611 |
| 3 | 0.200 | 0.021 | -0.046 | 0.599 | -0.045 | 0.608 | 0.137 | 0.118 | 0.000 | 1.000 |
| 4 | 0.072 | 0.453 | -0.014 | 0.887 | -0.050 | 0.607 | 0.221 | 0.020 | 0.010 | 0.918 |
| 5 | 0.459 | 0.000 | -0.033 | 0.760 | -0.110 | 0.307 | 0.361 | 0.001 | -0.037 | 0.732 |
| 6 | 0.160 | 0.200 | -0.069 | 0.579 | 0.370 | 0.002 | 0.082 | 0.510 | -0.053 | 0.675 |
| 7 | -0.029 | 0.853 | 0.067 | 0.667 | 0.237 | 0.121 | 0.097 | 0.531 | -0.080 | 0.605 |
| 8 | -0.046 | 0.841 | -0.044 | 0.847 | 0.117 | 0.604 | -0.091 | 0.687 | 0.273 | 0.219 |
| Selected lag length | 3 | | 0 | | 0 | | 1 | | 0 | |

All tests were significant at the 0.05 level.
